# Work Satisfaction and Post-traumatic Stress Disorder in Pediatric Healthcare Workers During the COVID-19 Pandemic Era

**DOI:** 10.7759/cureus.76726

**Published:** 2025-01-01

**Authors:** Nikolaos Rigas, Evangelia Antoniou, Eirini Orovou, Michael Kourakos, Alexandros Papatrehas, Zacharias Kyritsis, Maria Tzitiridou-Chatzopoulou

**Affiliations:** 1 Midwifery, University of West Attica, Athens, GRC; 2 Midwifery, University of Western Macedonia, Volos, GRC; 3 Nursing, School of Health Sciences, University of Ioannina, Ioannina, GRC; 4 Research, Non-Profit/Non Governmental Organization (NGO) "Fainareti", Athens, GRC; 5 Mathematics, Aristotle University of Thessaloniki, Thessaloniki, GRC; 6 Midwifery, University of Western Macedonia, Thessaloniki, GRC

**Keywords:** covid-19, job satisfaction, mental health, nurses, ptsd

## Abstract

Background/objectives

The COVID-19 pandemic has significantly affected the world, influencing various aspects beyond health-related issues. However, pediatric healthcare professionals experiencing anxiety due to COVID-19 exhibited elevated levels of work-related stress, were more likely to contemplate leaving their positions, and frequently reported low job satisfaction and higher rates of post-traumatic stress disorder (PTSD) symptoms.

The purpose of this study is to assess job satisfaction and work-related psychological trauma leading to PTSD in pediatric healthcare workers in Greek hospitals. The specific objectives also included examining the impact of intrinsic and extrinsic job satisfaction during the pandemic, alongside the prevalence of PTSD among pediatric healthcare workers, the relationship between job satisfaction and PTSD, and the correlation of the pandemic with PTSD stemming from personal, familial, or professional exposure to the fear of death caused by the COVID-19 era.

Methods

This cross-sectional study took place from October 2021 to June 2022 in pediatric wards, pediatric intensive care units (PICUs), and pediatric emergency departments of seven public university hospitals in Greece. The study included 445 pediatric healthcare workers, including physicians, nurses, and nursing aides.

Results

Our study revealed that 25.2% of pediatric healthcare professionals showed signs of post-traumatic symptoms. Furthermore, pediatric healthcare professionals who perceived a threat to their own lives or the lives of their loved ones due to COVID-19 were at a higher risk of developing PTSD compared to those who did not experience such fears. Our findings also indicated that higher levels of job satisfaction are linked to a lower risk of developing posttraumatic symptoms. Additionally, healthcare professionals who had been infected with COVID-19 reported lower job satisfaction levels compared to those who had not contracted the virus.

Conclusions

The elevated prevalence of PTSD among participants underscores the importance of implementing measures to safeguard and promote the mental well-being of staff in pediatric units.Moreover, improving job satisfaction is considered crucial, given its reciprocal relationship with the development of PTSD. We suggest conducting regular mental health evaluations for healthcare workers, ensuring sufficient rest periods, providing incentives for career advancement, optimizing the utilization of their skills and specialties, offering support from mental health professionals when symptoms are identified, and allowing the option of departmental transfers for those who desire it or show signs of mental illness.

## Introduction

The COVID-19 pandemic has had a profound impact globally, extending far beyond health concerns. It has significantly altered daily life and routines across various sectors, such as travel restrictions, sporting events, education disruptions, and beyond health concerns. During the pandemic, healthcare workers faced unique stressors in both their personal and professional lives, which could significantly influence their job satisfaction and decisions about staying in their roles [1]. Healthcare workers dealing with anxiety caused by COVID-19 displayed heightened work-related stress and a stronger tendency to consider leaving their jobs and very often reported increased burnout levels [2].

Job satisfaction among healthcare workers during the COVID-19 pandemic played a crucial role in influencing their performance, productivity, and retention rates. Job satisfaction is characterized by the relationship between professional expectations and rewards, primarily reflecting employees' attitudes, beliefs, knowledge, emotions, behaviors, and judgments [3]. Healthcare workers with lower levels of job satisfaction were at greater risk of experiencing burnout, diminished job performance [4], and an increased probability of leaving the profession. Additionally, dissatisfaction in their roles could adversely affect their mental and physical health, intensifying stress, anxiety, and other related conditions [5]. Furthermore, since pediatric healthcare workers come into direct contact with children suffering from injuries, accidents, illnesses, and their parents, the appearance of post-traumatic symptoms is likely [6].

It has been suggested that the symptoms of post-traumatic stress disorder (PTSD) fall into four categories: a) intrusion, b) avoidance, c) negative changes in cognition and mood, and d) alterations in arousal and reactivity, according to Diagnostic and Statistical Manual (DSM-5) [7]. Recently, a dissociative subtype of PTSD has also been included, which encompasses symptoms of depersonalization, derealization, and a high prevalence of comorbidity and symptom severity [7]. A necessary prerequisite, of course, is exposure to a traumatic experience, which is referred to as criterion A [8]. PTSD symptoms related to work in hospital environments can have significant consequences for healthcare workers and the overall healthcare system. For healthcare professionals, especially those in pediatric settings, the constant exposure to traumatic events, high-stress situations, and the emotional toll of caring for critically ill patients can lead to burnout and PTSD. These conditions can not only impair the workers' mental and physical health but also affect their job performance, increasing absenteeism, reducing productivity, and even leading to higher turnover rates [6]. The prevalence of PTSD among healthcare workers in critical departments reaches 30%, much higher than in the general population (3.5%) [9]. Since pediatric emergencies are often considered particularly psychologically taxing, healthcare professionals working in pediatrics may be at an increased risk of developing PTSD. Moreover, healthcare staff frequently delivers medical or nursing care without having had the chance to process stressful events or prioritize their own well-being, given that pre-existing anxiety disorders and depression are known risk factors for PTSD [10]. In addition, the appearance of COVID-19 in children, although generally mild during the first wave, proved to be particularly severe in children with underlying conditions, with several reports of burnout among pediatric workers (doctors and nurses) [10].

Furthermore, many healthcare workers contracted COVID-19, with some tragically losing their lives. However, during the pandemic, pediatricians and nurses faced increased workloads, challenges working in protective gear, fear of being infected or infecting their families, concerns about not being able to adequately assist patients, and issues such as fatigue, exhaustion, social isolation, stigma, and PTSD symptoms. In a study by Kackin [11], nurses expressed that they avoided social environments due to societal stigmatization and the risk of disease transmission, leading them to feel isolated and overwhelmed.

On the other side, low job satisfaction has been found to negatively affect an individual's psychosocial well-being [12]. Research indicates that anxiety is a major factor in lost workdays and is responsible for 50-60% of all such absences [13]. This underscores the severe impact of anxiety in the workplace. Anxiety becomes detrimental when it triggers physical and emotional responses arising from a mismatch between job demands and employees’ skills, available resources, or personal needs [14].

This discontent, subsequent anxiety, and PTSD symptoms can exhaust an individual's mental coping abilities, resulting in fatigue, somatization (physical symptoms without a clear medical explanation), and withdrawal from social interactions. These symptoms can then impact other areas of an individual's life, including their relationships with family and friends, their effectiveness as a parent, their academic endeavors, and their self-care routines [15]. Particularly in the realm of pediatric healthcare, research has indicated that working in high-stress settings such as pediatric intensive care units and pediatric emergency departments can intensify job dissatisfaction, resulting in burnout syndrome and secondary traumatic stress [16]. These departments are known for their treatment of young patients with life-threatening conditions and the significant levels of morbidity and mortality they encounter. The additional stress of addressing the emotional needs of parents also adds to the burden on healthcare professionals in these environments [17].

Since the COVID-19 period, the mental health of healthcare professionals has been tested, resources have been reduced, and decreased job satisfaction has resulted in a reduction of doctors and nurses in the public health system. At the same time, the fear of a new pandemic creates the need to strengthen the workforce by taking measures to address these working conditions, especially for pediatric healthcare workers. Therefore, the purpose of this study is to assess job satisfaction and work-related psychological trauma leading to PTSD in pediatric healthcare workers in Greek hospitals during the COVID-19 pandemic era. The following specific objectives were established: a) to determine the prevalence of PTSD among pediatric healthcare workers and assess the levels of intrinsic and extrinsic job satisfaction during the pandemic, b) to examine the relationship between job satisfaction and PTSD among pediatric healthcare workers during the pandemic, c) to investigate the impact of the pandemic on the development of PTSD stemming from personal, familial, or professional exposure to the fear of death caused by the COVID-19 era and finally, and d) the extent of the pandemic's impact on the job satisfaction of pediatric healthcare workers.

The specific objectives also included examining the impact of intrinsic and extrinsic job satisfaction during the pandemic, alongside the prevalence of PTSD among pediatric healthcare workers, the relationship between job satisfaction and PTSD, and the correlation of the pandemic with PTSD stemming from personal, familial, or professional exposure to the fear of death caused by the COVID-19 era.

## Materials and methods

This cross-sectional study was conducted between October 2021 and June 2022 in the pediatric wards, PICUs, and pediatric emergency departments of seven public university hospitals across Greece. Each hospital provided three pediatric specialties with equivalent levels of care. The survey involved pediatric healthcare professionals, including doctors, nurses, and nursing assistants, from the aforementioned university hospitals in Greece. Out of the 760 eligible healthcare workers across the seven university hospitals (with a total of 21 departments), 600 met the study criteria and agreed to participate by receiving and completing the questionnaires after being fully briefed by the researcher. Of these, 445 (75%) returned their completed questionnaires. To conduct this study, a multidisciplinary team collaborated, comprising mental health specialists, a pediatrician, and nurses from three university institutions and a mental health structure in Greece. The professionals were approached by the researcher in person during a break from their work. Then, they were informed about the purpose of the study, and after giving their consent, they were provided with the questionnaires, which were completed in a designated area that ensured their privacy.

Inclusion and exclusion criteria

To qualify for participation, they were required to have worked in their respective departments for at least one year. Additionally, participants needed to possess adequate proficiency in the Greek language to comprehend the questions in the psychometric tools. Full-time employment and a permanent contract with the hospital were also necessary conditions. Therefore, the exclusion criteria included insufficient knowledge of the Greek language and individuals who had worked in the respective department or unit for less than one year. However, due to necessary staff transfers between various departments because of illness or other needs, a significant number of workers were excluded. 

Measures

Data collection occurred after the researcher met with the pediatric healthcare workers, provided them with relevant information, and obtained their written consent to participate in the study. These meetings were held either during their work breaks or after their shifts had ended. The researcher oversaw the entire data collection process.

Socio-Demographic Questionnaire

A custom-designed questionnaire consisting of 21 questions was used to gather personal, social, demographic, and professional information (Appendix A).

Minnesota Satisfaction Questionnaire (MSQ) - Short Form [18]

This 20-item tool assesses the level of job satisfaction. Developed by the University of Minnesota in 1977, it is available in multiple languages, including Greek (Appendix B), and can be freely accessed for research purposes. The MSQ offers more detailed insights into specific aspects of a job compared to other measures and also examines the professional needs of employees. The short version of the MSQ takes approximately five minutes to complete. The MSQ provides more comprehensive information compared to other similar measures and also addresses the professional needs of healthcare workers and other employees. 

Post-traumatic Stress Disorder Checklist for DSM-5 (PCL-5) [19]

The PCL-5 is a self-administered questionnaire consisting of 20 items designed to assess PTSD symptoms over the past month based on DSM-5 criteria. Originally developed by the National Center for PTSD, the scale was translated and adapted into Greek (Appendix C) by Orovou et al. in 2021 [20], demonstrating excellent psychometric reliability (Cronbach’s α = 0.97). The PCL-5 can be scored in two main ways: a) a total score (ranging from 0 to 80) can be calculated by summing the scores of all 20 items, with higher scores indicating more severe PTSD symptoms, or b) specific diagnostic criteria can be evaluated, where any item scored two or above (“moderately”) is considered indicative of pathology. For instance, criteria B (questions 1-5) requires one or more pathological responses, criteria C (questions 6-7) requires one or more, criteria D (questions 8-14) requires two or more, and criteria E (questions 15-20) also requires two or more. In this study, the second scoring method was employed to assess symptom clusters alongside determining the presence or absence of the disorder. This approach provides a provisional PTSD diagnosis. The PCL-5 represents the latest DSM edition and is considered the most appropriate tool for measuring PTSD symptoms, particularly after the identification of trauma exposure, which, in our research, referred to exposure to life-threatening trauma during the COVID-19 pandemic in a pediatric work environment.

The study was conducted by the Declaration of Helsinki and approved by the (1) Children’s Hospital Agia Sofia in Athens Ethics Commission: 14972/30-07/2021, (2) “Pan. & Aglaia Kyriakou” Children’s Hospital in Athens Ethics Commission: 12652/14-07-2021, (3) Children’s Hospital in Athens, Attikon University General Hospital in Athens Ethics Commission: 6/7-7-2021, (4) General Children Hospital of Penteli in Athens Ethics Commission: 6425/25-06-2021, (5) General Hospital Hippokration, in Thessaloniki Ethics Commission: 23566/23-09-2021, (6) General University Hospital of Patras Ethics Commission: 330/06-07-2021, and (7) University General Hospital of Heraklion “PAGNI” in Crete Ethics Commission: 16246/21-9-21. The ethics committees examined information on specific ethical issues, such as participant anonymity, data handling, and the informed consent process beyond written consent.

Statistical analysis

Statistical analysis was conducted using SPSS software (IBM SPSS Statistics for Windows, IBM Corp., Version 27, Armonk, NY). Initially, descriptive statistics (mean and standard deviation) were calculated for all variables. The percentage (%) of pediatric healthcare workers who developed PTSD after exposure to COVID-19 was recorded. Furthermore, a forward analysis was conducted to highlight the relationship between demographic factors, work-related aspects, job satisfaction, and exposure to COVID-19 with the occurrence of PTSD. Subsequently, for the variables identified as significant from the chi-square independence test, logistic regression analysis was performed to estimate the risk of PTSD among workers during the COVID-19 period and to calculate the risk of PTSD based on levels of job satisfaction.

## Results

The threat of COVID-19

From the total of 445 healthcare workers in pediatric departments who participated in the survey, 67.4% (n = 300) reported feeling threatened for their own lives or the lives of their loved ones during the COVID-19 pandemic, and 77.3% (n = 344) reported that a relative or friend had contracted COVID-19. Additionally, 22.7% (n = 101) reported that they themselves had contracted COVID-19, and 21.3% (n = 95) reported that a relative or friend had died from COVID-19. Furthermore, from the total number of healthcare workers in pediatric departments, 60% (n = 267) reported having cared for a child with COVID-19, and 8.8% (n=39) reported that a child they had cared for had died from COVID-19 (Table 1).

**Table 1:** Results for various events related to COVID-19

	Yes		No	
	n	%	n	%
During the COVID-19 pandemic, did you feel a threat to your life or the lives of your loved ones?	300	67.4%	145	32.6%
Have you contracted COVID-19?	101	22.7%	344	77.3%
Has a relative or friend of yours contracted COVID-19?	344	77.3%	101	22.7%
Has a relative or friend of yours died from COVID-19?	95	21.3%	350	78.7%
Have you cared for a child with COVID-19?	267	60.0%	178	40.0%
Has a child you cared for died from COVID-19?	39	8.8%	406	91.2%

Work satisfaction

Overall, it appears that the mean score on the intrinsic satisfaction scale is 40.92 (SD = 6.77) on a scale ranging from 12 to 60. Additionally, the mean score on the extrinsic satisfaction scale is 18.38 (SD = 4.48) on a scale ranging from six to 30. The results indicate that, generally, employees in pediatric departments have a moderate to high level of intrinsic and extrinsic satisfaction. The average score for overall job satisfaction was found to be 69.6 (SD = 12.3). The majority of employees in pediatric departments had a mean score on a scale higher than 60 (corresponding to a moderate level of satisfaction) (Table 2).

**Table 2:** Results from descriptive analysis

	M	SD
Intrinsic satisfaction	40.9	6.8
Extrinsic satisfaction	18.4	4.5
Total satisfaction	69.5	12.4

PTSD

Table 3 below shows the percentages for each criterion met by individuals with and without PTSD.

**Table 3:** PTSD criteria PTSD, post-traumatic stress disorder

PTSD criteria	Participants who do not meet the criterion		Participants who meet the criterion	
	n	%	n	%
A sense of re-experiencing the traumatic event	229	51.5%	216	48.5%
Avoiding situations that remind you of the traumatic experience	248	55.7%	197	44.3%
Showing negative emotions about the traumatic event	260	58.4%	185	41.6%
Increased arousal and reactivity	247	55.5%	198	44.5%

**Figure 1:**
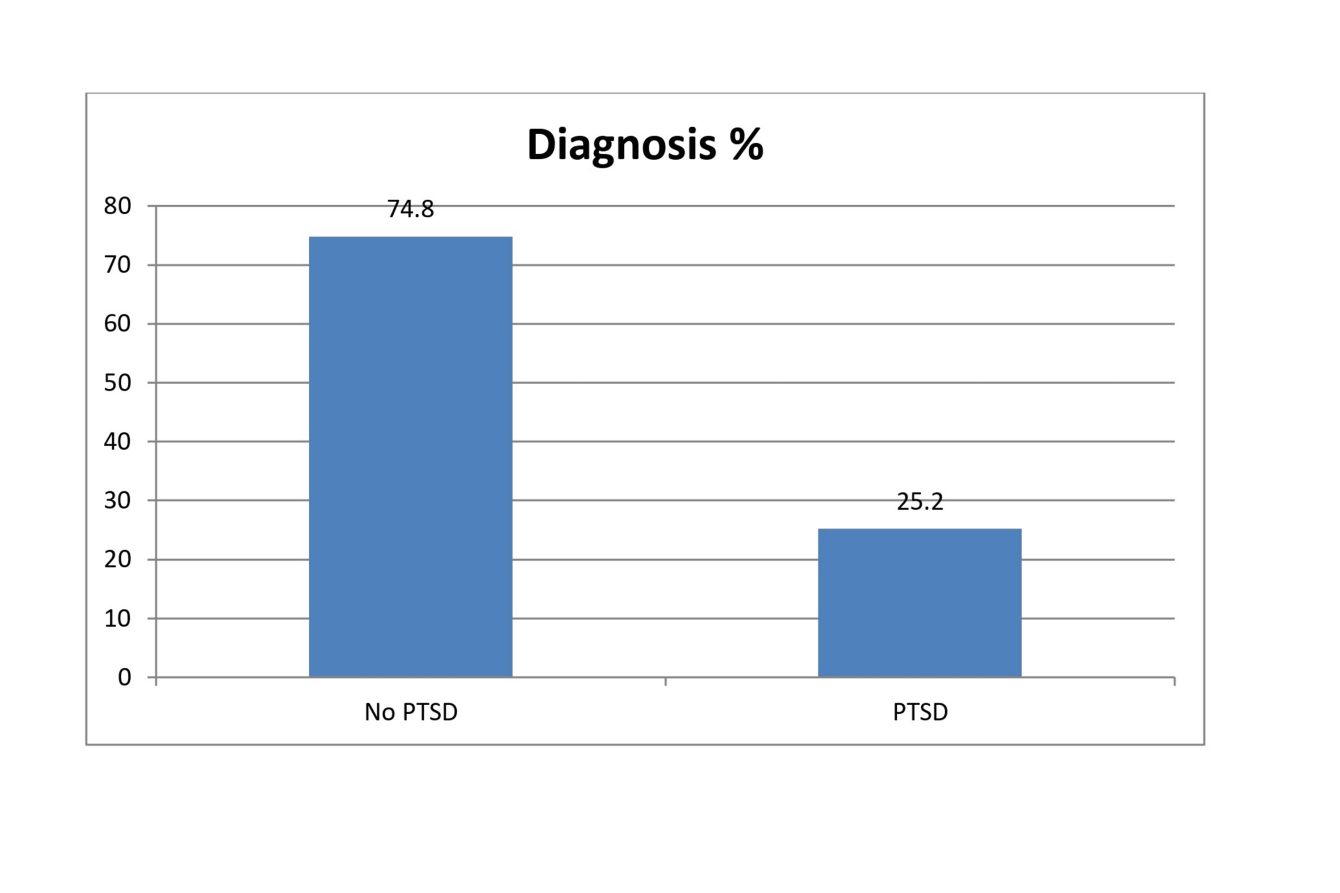
Results showing the percentage of workers in pediatric fields experiencing post-traumatic stress disorder (PTSD)

The results also show in Figure 1 that 25.2% (n = 112) of the participants exhibit characteristics indicative of a diagnosis of PTSD.

PTSD and COVID-19

The results of the multivariable logistic regression analysis (Table 4) indicate that workers in pediatric fields who have felt a threat to their own lives or the lives of their loved ones are 2.961 times more likely to develop post-traumatic stress compared to those who have not experienced such a threat (B = 1.086, p < 0.001, Exp(B) = 2.961, 95% CI (1.707, 5.136)).

**Table 4:** Results of multivariate logistic regression with the dependent variable being the presence of post-traumatic stress disorder (PTSD) and independent variables related to COVID-19 df, degrees of freedom; reference groups, no

	B	Standard error	df	p	Exp(B)	95% confidence interval	
						Lower	Upper
Feeling of threat to life	1.086	.281	1	0.000	2.961	1.707	5.136
Illness	-0.004	.271	1	0.988	.996	0.586	1.694
Illness of a relative/friend	-0.493	.276	1	0.074	.611	0.356	1.048
Death of a relative/friend	0.319	.273	1	0.243	1.376	0.805	2.351
Child hospitalization	-0.141	.232	1	0.544	.868	0.551	1.370
Child death	0.141	.380	1	0.710	1.152	0.547	2.426
Stable	-1.507	.315	1	0.000	.222	-	-

Work satisfaction and COVID-19

The findings from Table 5 indicate statistically significant differences in overall job satisfaction levels depending on whether pediatric healthcare workers had experienced a perceived threat to their lives or the lives of their loved ones (t(440) = -3.833, p = 0.000), had contracted COVID-19 (t(440) = -2.128, p = 0.034), or had encountered the death of a child due to COVID-19 in their workplace (t(440) = -2.134, p = 0.033). Workers who had experienced a perceived threat to their lives or the lives of their loved ones (M = 68.0, SD = 12.3) exhibited lower levels of overall job satisfaction compared to those who had not (M = 72.7, SD = 12.2). Workers who had contracted COVID-19 (M = 67.2, SD = 13.2) reported lower levels of overall satisfaction compared to those who had not contracted the virus (M = 70.2, SD = 12.1). Finally, workers who had encountered the death of a child due to COVID-19 in their workplace (M = 65.5, SD = 11.3) demonstrated lower satisfaction levels than those who had not experienced such a death (M = 69.9, SD = 12.4).

**Table 5:** Comparison of overall satisfaction in relation to COVID-19-related events

	Yes		No		t	p
	M	SD	M	SD		
Feeling of threat to life	40.1	6.7	42.5	6.7	-3.642	0.000
Illness	39.4	7.4	41.3	6.5	-2.544	0.011
Illness of a relative/friend	41.0	6.7	40.6	7.2	0.525	0.600
Death of a relative/friend	41.2	6.8	40.8	6.8	0.592	0.554
Child hospitalization	40.8	6.8	40.8	6.8	-0.428	0.669
Child death	39.7	6.4	41.0	6.8	-1.167	0.244

From Table 6, it appears that there is a statistically significant difference in intrinsic satisfaction levels based on whether healthcare workers in pediatric fields felt a threat to their own lives or the lives of their loved ones (t(440) = -3.642, p = 0.000) and whether they had been infected with COVID-19 (t(440) = -2.128, p = 0.034). Workers who felt a threat to their lives or the lives of their loved ones (M = 40.1, SD = 6.7) reported lower levels of intrinsic satisfaction compared to those who did not feel such a threat (M = 42.5, SD = 6.7). Similarly, workers who had been infected with COVID-19 (M = 39.4, SD = 7.4) showed lower levels of intrinsic satisfaction compared to those who had not been infected (M = 41.3, SD = 6.5).

**Table 6:** Comparison of intrinsic satisfaction in relation to COVID-19-related events

	Yes		No		t	p
	M	SD	M	SD		
Feeling of threat to life	40.1	6.7	42.5	6.7	-3.642	0.000
Illness	39.4	7.4	41.3	6.5	-2.544	0.011
Illness of a relative/friend	41.0	6.7	40.6	7.2	0.525	0.600
Death of a relative/friend	41.2	6.8	40.8	6.8	0.592	0.554
Child hospitalization	40.8	6.8	40.8	6.8	-0.428	0.669
Child death	39.7	6.4	41.0	6.8	-1.167	0.244

From Table 7, it appears that there is a statistically significant difference in the level of extrinsic satisfaction based on whether healthcare workers in pediatric fields felt a threat to their own lives or the lives of their loved ones (t(440) = -3.055, p = 0.002), whether they had experience with a child hospitalized with COVID-19 (t(440) = -3.105, p = 0.002), and whether they had experienced the death of a child from COVID-19 in their workplace (t(440) = -2.613, p = 0.009).

**Table 7:** Comparison of extrinsic satisfaction with COVID-19-related events

	Yes		No		t	p
	M	SD	M	SD		
Feeling of threat to life	17.9	4.5	19.3	4.4	-3.055	0.002
Illness	17.9	4.5	18.5	4.5	-1.197	0.232
Illness of a relative/friend	18.5	4.5	18.0	4.6	1.021	0.308
Death of a relative/friend	18.5	4.8	18.3	4.4	0.320	0.749
Child hospitalization	17.8	4.5	19.2	4.4	-3.105	0.002
Child death	16.6	4.1	18.5	4.5	-2.613	0.009

Workers who felt a threat to their lives or the lives of their loved ones (M = 17.9, SD = 4.5) reported lower levels of extrinsic satisfaction compared to those who did not feel such a threat (M = 19.3, SD = 4.4). Workers who had experience with a child hospitalized with COVID-19 (M = 17.8, SD = 4.5) showed lower levels of extrinsic satisfaction compared to those who did not have such experience (M = 19.2, SD = 4.4). Finally, workers who had experienced the death of a child from COVID-19 in their workplace (M = 16.6, SD = 4.1) showed lower levels of extrinsic satisfaction compared to those who had not experienced the death of a child from COVID-19 in their workplace (M = 18.5, SD = 4.5).

Work satisfaction and PTSD

The t-test revealed a statistically significant difference in the mean values of overall job satisfaction (t(440) = 6.597, p < 0.001), intrinsic satisfaction (t(440) = 5.614, p < 0.001), and extrinsic satisfaction (t(440) = 6.143, p < 0.000) based on whether healthcare workers in pediatric fields have PTSD or not. The analysis showed that healthcare workers in pediatric fields who meet all the criteria for PTSD (M = 63.13, SD = 11.62) have lower levels of professional satisfaction compared to those who do not meet the criteria for PTSD (M = 71.66, SD = 11.90). Similarly, healthcare workers in pediatric fields who meet all the PTSD criteria (M = 37.87, SD = 6.82) have lower levels of intrinsic job satisfaction compared to those who do not meet the PTSD criteria (M = 41.89, SD = 6.47). Finally, healthcare workers in pediatric fields who meet all the PTSD criteria (M = 16.18, SD = 4.05) have lower levels of extrinsic job satisfaction compared to those who do not meet the PTSD criteria (M = 19.08, SD = 4.42) (Table 8).

**Table 8:** Comparison of job satisfaction based on the presence of PTSD PTSD, post-traumatic stress disorder

	Without PTSD		PTSD		t	p
	M	SD	M	SD		
Work satisfaction	71.7	11.9	63.1	11.6	6.579	<0.001
Intrinsic satisfaction	41.9	6.5	37.9	6.8	5.614	<0.001
Extrinsic satisfaction	19.1	4.4	16.2	4.0	6.143	<0.001

In Table 9, the findings regarding the prediction of the risk of developing PTSD based on levels of job satisfaction are presented. The results show that only extrinsic satisfaction is a significant risk factor for developing PTSD (B = -0.112, p < 0.05, Exp(B) = 0.894, 95% CI (0.833, 0.960)). The findings indicate that an increase of one unit in the extrinsic satisfaction score is associated with a 10.6% decrease in the likelihood of developing PTSD.

**Table 9:** Multivariate logistic regression to predict the risk of developing PTSD based on job satisfaction levels of employees in pediatric fields df, degrees of freedom

		B	Standard error	df	p	Exp(B)	95% confidence interval	
							Lower	Upper
Step 1	Intrinsic	-0.042	0.023	1	0.071	0.959	0.916	1.004
	Extrinsic	-0.112	0.036	1	0.002	0.894	0.833	0.960
	Constant	2.570	0.712	1	0.000	13.071	-	-

## Discussion

According to the results of our study, 25.2% of pediatric healthcare workers exhibited post-traumatic symptoms, and these findings are consistent with the international literature demonstrating a high exposure to psychological trauma during their work, in contrast to the general population, where PTSD has a prevalence rate of 3.5% [9]. Based on these findings, pediatric healthcare workers constitute a high-risk group for developing post-traumatic symptoms. 

Moreover, the COVID-19 period brought numerous challenges to people's lives, with an increase in anxiety, depression, and other mental health disorders [21] as a result of specific living conditions, lockdowns, and the fear of death. Although anyone could experience mental or emotional difficulties related to COVID-19, certain groups, such as healthcare workers, appeared to be at an increased risk of developing psychopathological disorders during or after the pandemic [22]. According to our results, pediatric healthcare workers who had felt a threat to their own or their loved ones' lives due to COVID-19 were more likely to develop PTSD compared to those who did not feel such a threat to their lives or their loved ones, and this reflects the exposure to the fear of death, as a necessary condition for the development of PTSD. Indeed, although healthcare professionals often face death and injury as part of their daily work, the pandemic introduced additional challenges, such as increased uncertainty, threats to their personal safety, concerns about colleagues contracting the virus, and a higher patient load. Furthermore, the literature highlights a strong correlation between perceived risk and the development of PTSD, even under pandemic conditions [23]. Moreover, The daily exposure of pediatric healthcare workers to psychological trauma involving children increases the risk of developing PTSD, particularly when caring for children in the terminal stages of an illness. Additionally, they must cope with the loss, which may trigger their past traumas, and the unbearable grief of the parents, a burden that becomes especially overwhelming for pediatric healthcare workers who are parents themselves [6]. Pediatric healthcare workers, therefore, constitute a vulnerable healthcare population to psychological trauma.

Our results also showed that increased work satisfaction is associated with a reduced likelihood of developing PTSD. In general, the international literature emphasizes the positive factors that can protect individuals from traumatic events, such as personal resilience, life satisfaction, and gratitude are considered some of these protective factors [24]. Job satisfaction, although not widely investigated in relation to the development of post-traumatic symptoms, appears to be an important predictive factor for the development of PTSD. Specifically, Kelly and Lefton (2017) [25] found that by increasing job satisfaction, post-traumatic symptoms in nurses were reduced. On the other hand, low job satisfaction has been linked to an increase in secondary PTSD in a group of nurses in China [26]. Additionally, a study on nurses in the United States showed a negative correlation between post-traumatic symptoms and job satisfaction, as well as a positive correlation between decreased job satisfaction and burnout [27].

The results of this study also indicated that healthcare workers who had contracted COVID-19 exhibited lower levels of job satisfaction compared to those who had not contracted the virus. It is a fact that the COVID-19 pandemic had a global impact, with changes in daily work routines leading to psychological strain on individuals, which may affect their performance and well-being in the workplace [28]. Healthcare professionals were exposed to work-related stress under normal conditions, both due to their interactions with patients and the difficult working conditions (long shifts and on-call duties, fatigue, insomnia, isolation from family and society, violence from patients, etc.) [29]. In addition to these, the intense stress caused by the COVID-19 pandemic led to burnout and, consequently, reduced job satisfaction and exhaustion [29]. 

Finally, the COVID-19 pandemic disrupted the daily routines of healthcare professionals worldwide. Despite the cultural differences in healthcare systems, the prevalence of PTSD and levels of job satisfaction appeared to align [25-29]. It seems that the unexpected nature of the pandemic and the threat to human life were shared experiences among all healthcare workers, as exposure to trauma and fear were common characteristics.

This study presented both strengths and limitations. A key strength was the sufficient sample size, which allowed for the extraction of reliable conclusions in many cases. Additionally, the variable of job satisfaction was used to identify its relationship with PTSD during the COVID-19 pandemic using specialized questionnaires. More specifically, the MSQ provides more comprehensive information compared to other similar measures and also addresses the professional needs of employees, while the PCL-5 represents the latest DSM edition and is considered the most appropriate tool for measuring PTSD symptoms, particularly after the identification of trauma exposure. One of the main limitations concerned the cross-sectional design of the study, which does not allow us to track the evolution of symptoms after the pandemic nor to identify the factors that may contribute to their improvement or worsening. More specifically, a prospective study could help us understand how changes in departments or the provision of psychological support affect the job satisfaction and PTSD symptoms of healthcare workers. Furthermore, given that the collected data were derived from self-reported questionnaires rather than clinical assessments, response bias is a possibility, as the reported symptoms or levels of job satisfaction may not accurately reflect their true experiences.

## Conclusions

This study identified a connection between job satisfaction and post-traumatic symptoms among pediatric healthcare professionals in public hospitals in Greece. The high rates of PTSD among participants highlight the need for measures that will protect and support the mental health of workers in pediatric departments. Furthermore, enhancing job satisfaction is deemed essential, as it is bi-directionally linked to the occurrence of PTSD. We recommend regular mental health assessments of healthcare workers, the provision of adequate rest time, the offering of incentives for professional development, optimizing the use of their skills and specialties, support from mental health professionals when symptoms are detected, and the option for department changes for those who wish to or exhibit signs of psychological trauma. The exchange of opinions and personal experiences regarding the traumatic event among colleagues appears to weaken the intensity of the trauma and is also recommended. In addition, recognizing the contributions of healthcare workers and providing opportunities for personal development will enhance work satisfaction levels and contribute to mental resilience and adaptation to mental trauma. Another important measure would be increasing the number of healthcare professionals, which should be seriously considered by decision-makers as part of the solution to the problem. Additionally, targeted studies should be conducted to assess other factors related to the development of PTSD, such as the role of professional burnout, as well as the effectiveness of interventions aimed at enhancing psychological resilience and reducing burnout symptoms, given that these parameters are predictors for the development of PTSD among pediatric workers. We also recommend future research that will examine how broader institutional or systemic factors (e.g., hospital policies, staffing levels, access to mental health resources) affect job satisfaction and, consequently, the risk of PTSD. Although the COVID-19 pandemic may have passed, preparedness for future pandemics remains imperative.

## Figures and Tables

**Figure 2:**
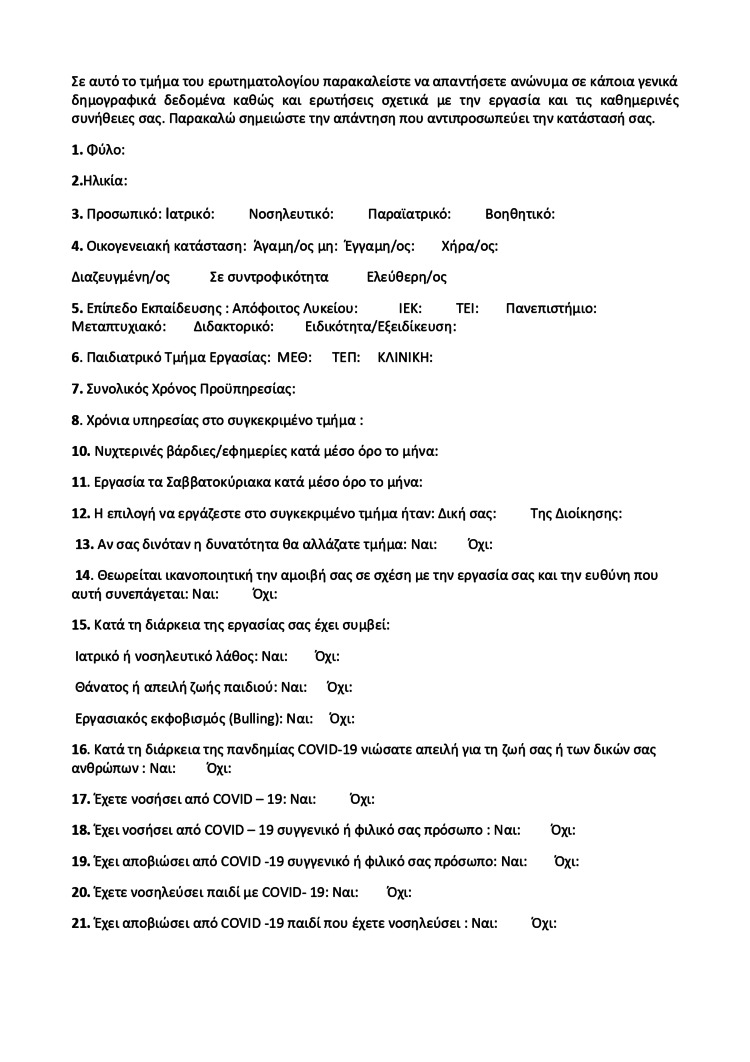
Demographics

**Figure 3:**
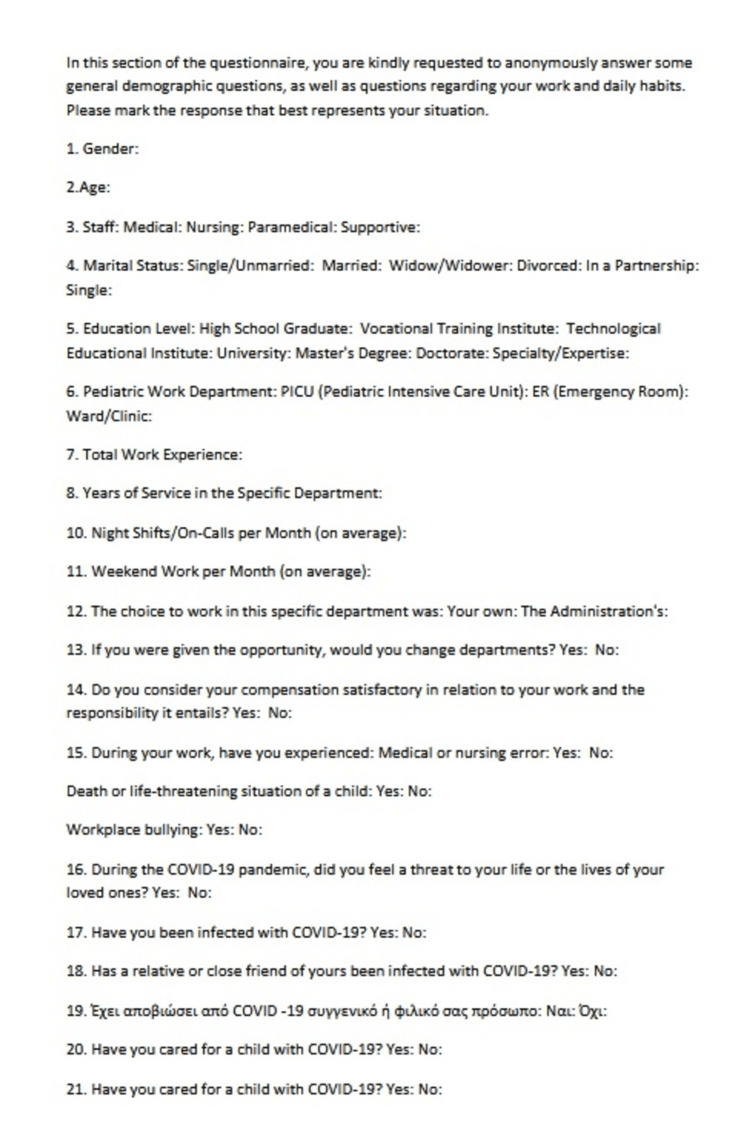
Demographics (English)

**Figure 4:**
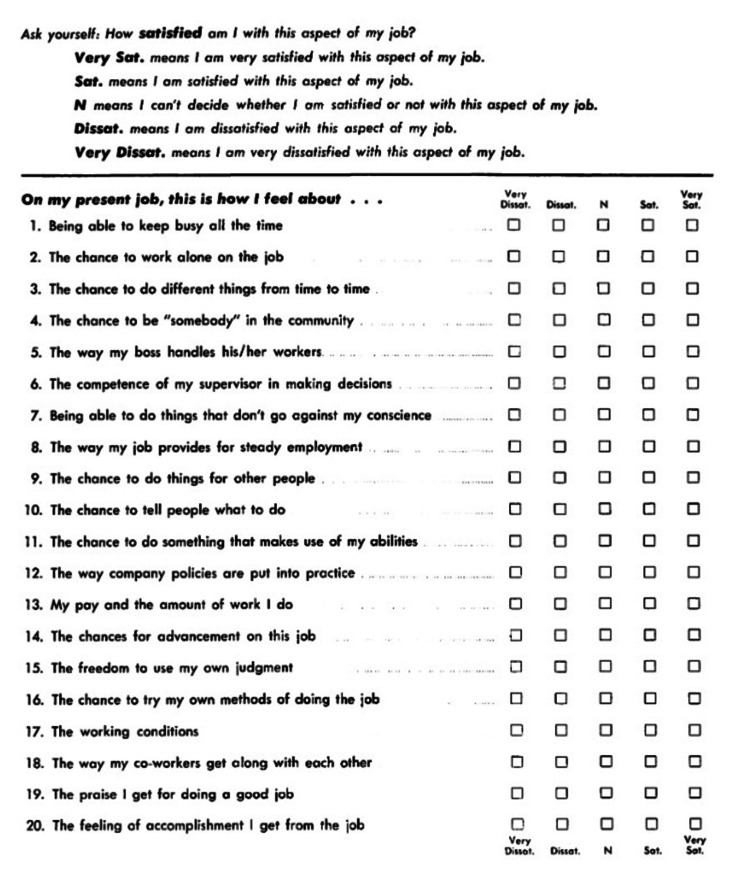
Minnesota Satisfaction Questionnaire (English)

**Figure 5:**
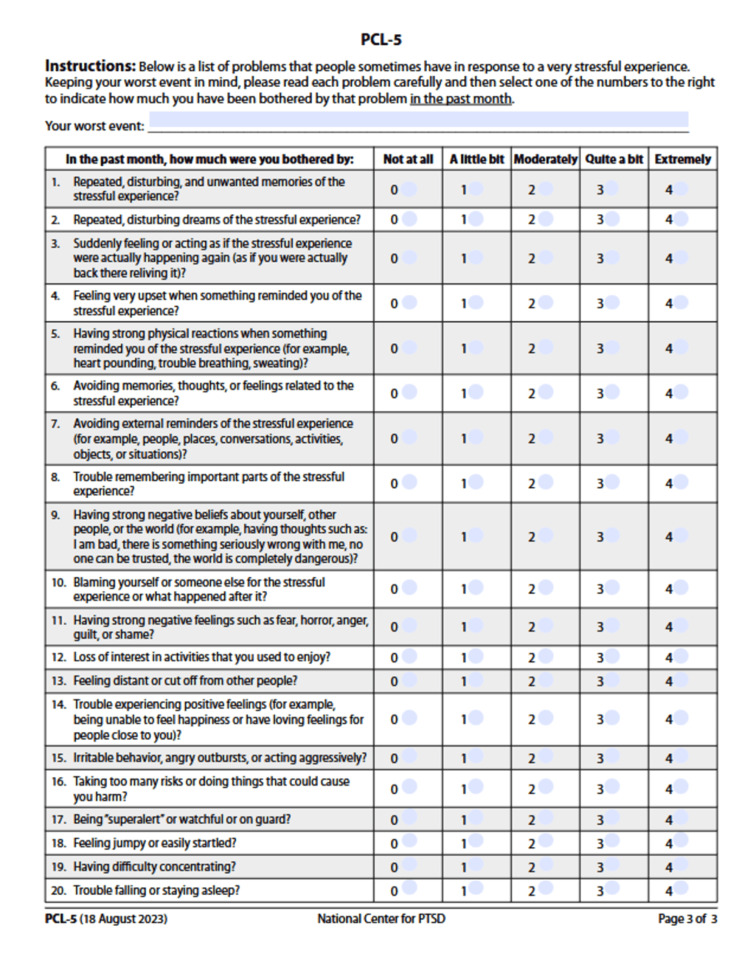
Post-traumatic Stress Disorder Checklist for DSM-5

## Appendices

Appendix A 

Appendix B 

**Table 10:** Minnesota Satisfaction Questionnaire

σχετικά με …	Πολύ Δυσαρ.	Δυσαρ.	Ουδέτ.	Ικανοπ.	Πολύ Ικανοπ.
1. τη δυνατότητα να μη μένω άνεργος.	1	2	3	4	5
2. τη δυνατότητα να είμαι ανεξάρτητος	1	2	3	4	5
3. την ευκαιρία να έχω ποικιλία δραστηριοτήτων.	1	2	3	4	5
4. την ευκαιρία να είμαι <> στο χώρο μου.	1	2	3	4	5
5. τον τρόπο που μου συμπεριφέρονται οι προϊστάμενοι μου.	1	2	3	4	5
6. την ικανότητα του προϊσταμένου μου να παίρνει αποφάσεις.	1	2	3	4	5
7. τη δυνατότητα να κάνω πράγματα σύμφωνα με τη συνείδησή μου.	1	2	3	4	5
8. τη δυνατότητα να έχω σταθερή απασχόληση.	1	2	3	4	5
9. την ευκαιρία να κάνω πράγματα για άλλους ανθρώπους.	1	2	3	4	5
10. την ευκαιρία να καθοδηγώ άλλους ανθρώπους.	1	2	3	4	5
11.την ευκαιρία να χρησιμοποιώ τα προσόντα μου.	1	2	3	4	5
12. τον τρόπο με τον οποίο ασκεί τη πολιτική της η διοίκηση	1	2	3	4	5
13. τον μισθό αναλογικά με τη δουλειά που κάνω.	1	2	3	4	5
14. τις ευκαιρίες για προαγωγή ή ανέλιξη.	1	2	3	4	5
15. την ελευθερία να χρησιμοποιώ τη δική μου κρίση.	1	2	3	4	5
16. την ευκαιρία να εφαρμόζω δικές μου μεθόδους/ιδέες.	1	2	3	4	5
17. τις εργασιακές συνθήκες.	1	2	3	4	5
18. τις σχέσεις των συναδέλφων μεταξύ τους.	1	2	3	4	5
19. την αναγνώριση που μου δίνουν.	1	2	3	4	5
20. το αίσθημα ολοκλήρωσης που εισπράττω.	1	2	3	4	5

Appendix C 

**Table 11:** Post-traumatic Stress Disorder Checklist for DSM-5

		Καθόλου	Λίγο	Μέτρια	Αρκετά	Πάρα πολύ
1	Επαναλαμβάνονται ανεπιθύμητες αναμνήσεις του τραυματικού γεγονότος που σας προκαλούν ανησυχία;	0	1	2	3	4
2	Επαναλαμβάνονται ενοχλητικά όνειρα που σχετίζονται με το τραυματικό γεγονός;	0	1	2	3	4
3	Αισθάνεστε και δράτε σαν να ξανασυμβαίνει το τραυματικό γεγονός και να το ζείτε;	0	1	2	3	4
4	Αναστατώνεστε και ταράζεστε όταν κάτι σας θυμίσει το τραυματικό γεγονός του παρελθόντος;	0	1	2	3	4
5	Νιώθετε πως έχετε περίεργες σωματικές αντιδράσεις όπως ταχυκαρδία και ιδρώτα κάθε φορά που σκέφτεστε το τραυματικό γεγονός που ζήσατε;	0	1	2	3	4
6	Αποφεύγετε αναμνήσεις, σκέψεις ή συναισθήματα που σχετίζονται με το συγκεκριμένο τραυματικό γεγονός;	0	1	2	3	4
7	Αποφεύγετε καταστάσεις ή δραστηριότητες που σας ξυπνούν αναμνήσεις και σας θυμίζουν το τραυματικό γεγονός;	0	1	2	3	4
8	Δυσκολεύεστε να θυμηθείτε σημαντικά στοιχεία του τραυματικού γεγονότος που ζήσατε;	0	1	2	3	4
9	Έχετε αρνητική πεποίθηση για τον εαυτό σας, για άλλους ανθρώπους και για τα γεγονότα που συμβαίνουν στον κόσμο; (πχ νιώθετε πως φταίτε για όλα, ότι δεν μπορείτε να εμπιστευτείτε κανέναν ή ότι ο κόσμος είναι επικίνδυνος;)	0	1	2	3	4
10	Κατηγορείτε τον εαυτό σας ή κάποιον άλλο για τη την τραυματική εμπειρία ή για το τι ακολούθησε;	0	1	2	3	4
11	Νιώθετε έντονα αρνητικά συναισθήματα όπως φόβο, τρόμο, θυμό, ενοχές ή ντροπή για το τραυματικό γεγονός που ζήσατε;	0	1	2	3	4
	Έχετε χάσει το ενδιαφέρον σας για δραστηριότητες που συνηθίζατε στο παρελθόν;	0	1	2	3	4
13	Νιώθετε απόμακρη/ος ή αποξενωμένη/ος από τα άτομα του περιβάλλοντος σας;	0	1	2	3	4
14	Νιώθετε συναισθηματική απάθεια ή έχετε δυσκολία να εκφράσετε συναισθήματα αγάπης προς τους κοντινούς σας ανθρώπους;	0	1	2	3	4
15	Νιώθετε πως έχετε ευερεθιστότητα, εκρήξεις θυμού ή έντονη επιθετικότητα;	0	1	2	3	4
16	Εκτίθεστε σε πολλούς κινδύνους ή κάνετε πράγματα που μπορούν να σας βλάψουν;	0	1	2	3	4
18	Νιώθετε να ξαφνιάζεστε εύκολα ή να τρομάζετε εύκολα;	0	1	2	3	4
